# Transforming multi-omics data into images for disease classification: A review of techniques and tools

**DOI:** 10.1016/j.jpi.2026.100543

**Published:** 2026-01-16

**Authors:** Ali Alyatimi, Muhammad Atif Iqbal, Vera Chung, Seid Miad Zandavi, Ali Anaissi

**Affiliations:** a Faculty of Engineering, School of Computer Science, The University of Sydney, Sydney, NSW 2008, Australia; b Department of Computer and Information Technology, Jazan College of Technology, Technical and Vocational Training Corporation (TVTC), Riyadh 12613, Saudi Arabia; c Harvard Medical School, Boston, MA, USA

**Keywords:** Multi-omics integration, Deep learning, Image-based omics transformation, Disease classification, Bioinformatics

## Abstract

The integration of multi-omics data has become crucial in understanding the complexity of biological systems and disease mechanisms. However, the high dimensionality and heterogeneity of such data present significant analytical challenges. This review investigates the emerging approach of transforming multi-omics non-image data into image formats to facilitate the application of advanced deep learning techniques for disease classification and biomarker discovery. This article presents a scoping review of studies published between 2013 and 2024, focusing on techniques that convert multi-omics data into images. Various transformation methods, including t-SNE, kernel PCA, UMAP, FFT, and treemaps, were examined alongside deep learning models such as convolutional neural networks, autoencoders, support vector machines, graph convolutional networks, and graph neural networks. The transformation of omics data into image formats enables effective feature extraction and classification, with reported accuracies ranging from 75% to 99% across various studies. CNN-based models, in particular, demonstrated superior performance in integrating complex molecular interactions. Despite these advances, challenges such as overfitting, limited generalizability, and interpretability persist, especially given the diversity and complexity of multi-omics datasets. Finally, the transforming multi-omics data into images represents a promising direction in biomedical research, facilitating more profound insights into disease mechanisms and improving predictive modeling. Addressing current limitations through improved model interpretability, robust transformation methods, and larger, more diverse datasets will be essential for realizing the full potential of this approach in precision medicine.

## Introduction

### Background

The terms “-ome” and “omics” are commonly used by biologists, especially in high-throughput experiments designed to understand molecular biology fields such as genomics, transcriptomics, proteomics, metabolomics, and metagenomics. These high-throughput approaches enable comprehensive profiling of molecular components, providing insights into how genetic, epigenetic, and metabolic factors collectively drive cellular function and disease processes.^1^^,^^2^ Moreover, the integration of these diverse data layers, referred to as multi-omics, ensures a clear understanding of biological mechanisms and is vital for biomedical and precision medicine research.^3^^,^^4^

Despite significant progress, several challenges hinder effective multi-omics integration. These include the heterogeneity and high dimensionality of data, variability across experimental platforms, and the lack of standardized computational frameworks capable of capturing complex molecular interactions. Addressing these challenges is critical for translating multi-omics insights into clinical applications such as biomarker discovery, disease stratification, and therapeutic target identification. Furthermore, the advances in computational modeling, particularly deep learning, offer advantageous techniques for handling and interpreting multi-omics data. Traditional statistical methods, including principal component analysis (PCA) and regression models, remain valuable for single-omics analysis. In contrast, they often may fail to capture the non-linear dependencies across multiple omics layers. In contrast, advanced integrative approaches such as matrix factorization, multi-view learning, and deep learning architectures can model complex, synergistic relationships among molecular datasets.^3^ An emerging direction involves transforming non-image omics data into image-based representations, enabling the use of convolutional neural networks (CNNs) and other computer vision models to enhance feature extraction and biological pattern recognition. Approaches such as DeepInsight demonstrate how these image-based transformations can reveal hidden biological relationships and improve diagnostic prediction and biomarker discovery.^5^

Ultimately, this review aims to synthesize current advances in multi-omics data integration, with a particular focus on deep learning and multi-view learning methodologies. It further explores recent developments in image-based data transformation techniques and their potential to enhance biological interpretation. By identifying current challenges and knowledge gaps, this review seeks to guide the development of more robust, interpretable, and unified frameworks for multi-omics analysis in biomedical research.

### Motivation and scope

This study seeks to explore the emerging trend of transforming multi-omics data into image formats. It is furthermore essential to evaluate methods that identify the most accurate and practical approaches in this field. The scope of this article includes a comprehensive review of transformation techniques and deep learning models.

## Research methodology

### Study selection

**Fig. 1:**
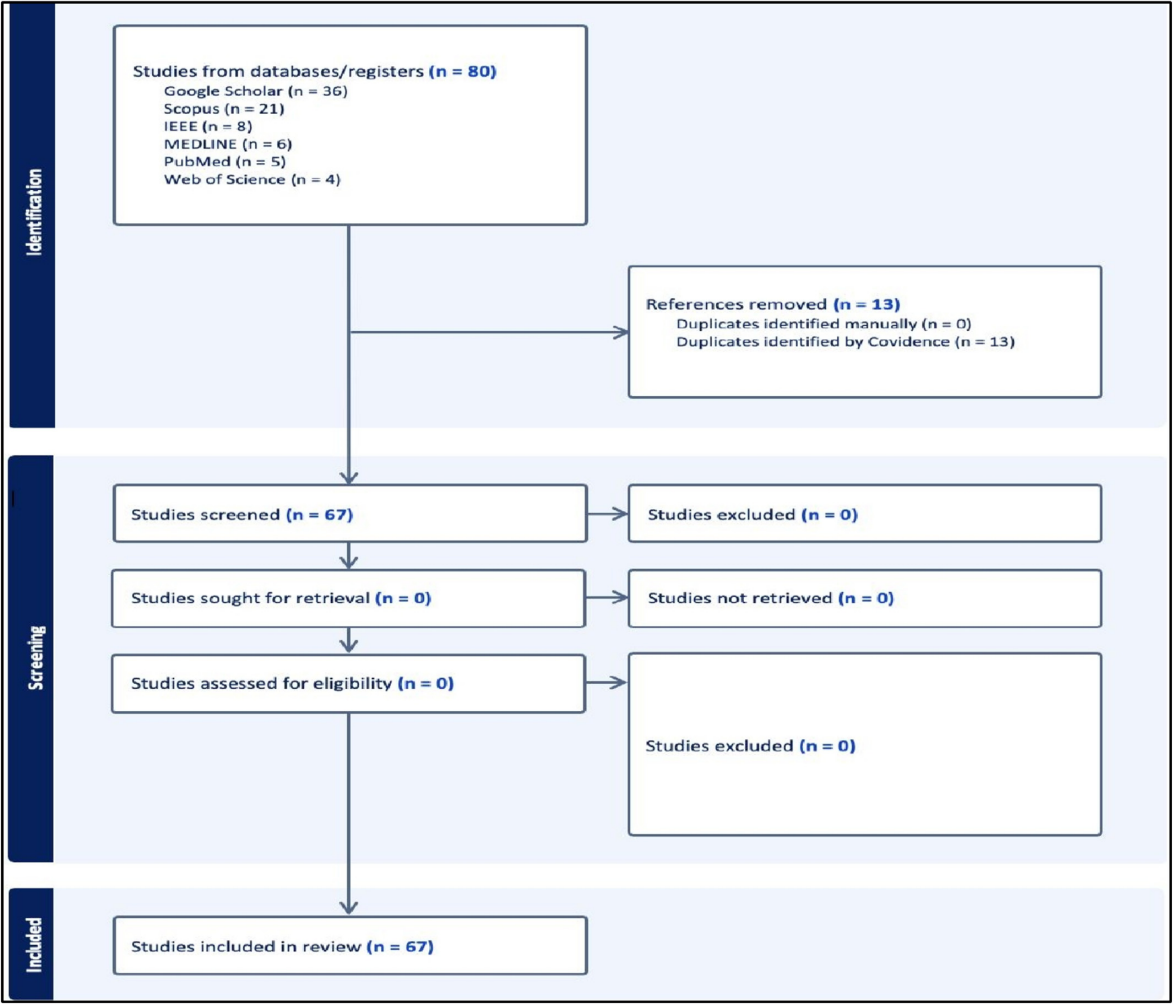
PRISMA flow diagram for the multi-omics scoping review conducted using Covidence.

The scoping review followed JBl protocols and reported per PRISMA-SCR (Fig. 1), a comprehensive search was conducted across 6 databases: Google Scholar, Scopus, IEEE Xplore, MEDLINE via Ovid, Web of Science, and PubMed, yielding a total of 80 studies (Fig. 1). Using Covidence, two independent reviewers systematically screened the literature. The platform automatically detected and removed 13 duplicate records, leaving 67 unique studies for title- and abstract-level assessment. Each record was then appraised against the a priori eligibility criteria by both reviewers, as depicted in the PRISMA flow diagram, yielding the final set of studies included in the review.

### Search strategy

The search strategy was constructed by combining key terms related to multi-omics data integration, deep learning, image transformation, and disease-related applications. Keyword selection was informed by exploratory searches in major databases, citation chaining from highly cited articles, and terminology used in relevant systematic reviews. To maximize coverage and relevance, combinations of specific concepts were used across multiple queries. The final set of search terms reflects common terminology in the field and was designed to capture a focused but comprehensive set of publications.

### Search criteria

• Integration of multi-omics data using deep learning or machine learning.
• Focus on disease classification, diagnosis, and biomarker discovery.
• Approaches converting omics data to image-like formats for analysis.
• Limited to peer-reviewed journal articles and reviews.
• Publication timeframe set between 2013 and 2024 to capture deep learning advancements in biomedical informatics.

### Search terms

• “Multi-omics integration” AND “deep learning”
• “Multi-omics integration” AND “deep learning” AND “disease diagnosis”
• “Omics data” AND “image processing” AND “disease classification”
• “Multi-omics image transformation” AND “machine learning”
• “Non image data to an image” AND “omics data”

## Research results

**Fig. 2:**
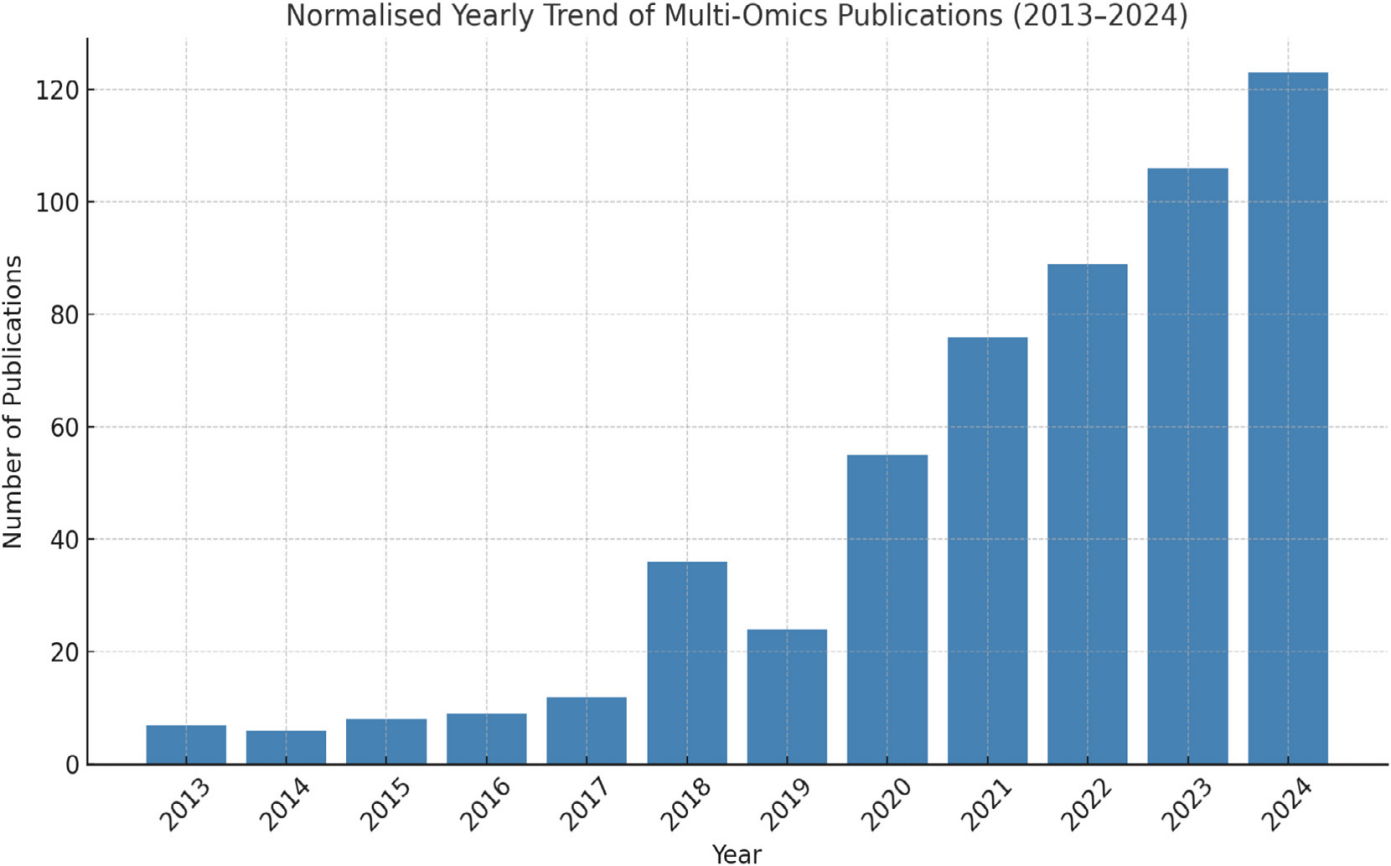
Normalized yearly trend of the “multi omics” keyword in Scopus© publications (2013–2024). The trend reflect the relative propotion of multi-omics research among all publications in the selected subject areas and document types, demonstrating a consistent increase over time.

The Scopus dataset used for this analysis includes research publications from 2013 to 2024, retrieved using the search term “multi-omic” in the article title. To ensure relevance to interdisciplinary biomedical research, the results were filtered by subject areas Biochemistry, Genetics, Molecular Biology, Medicine, and Computer Science and limited to journal articles and reviews. Fig. 2 presents the normalized yearly trend of multi-omics publications, calculated as a percentage of the total number of publications retrieved per year under the same filters. The results indicate a substantial relative increase in multi-omics research activity, rising from 1.3% in 2013 to over 22% in 2024. This reflects the growing integration of omics technologies and AI-driven computational approaches in biomedical research, underscoring the increasing importance of multi-omics analysis in disease understanding and precision medicine.

### Multi-omics data

Multi-omics refers to the integration of multiple high-throughput biological data types, such as genomics, transcriptomics, epigenomics, and proteomics, to gain a comprehensive understanding of biological systems and diseases. Traditional single-omics approaches, which primarily analyze a single type of molecular data, often limit insights into complex biological processes and are insufficient for understanding multifactorial diseases.^6^

Multi-omics enables researchers to uncover complex molecular interactions, classify diseases more accurately, and predict patient outcomes more precisely. For instance, Wei et al.^7^ emphasized that multi-omics data integration can reveal tumor heterogeneity and improve molecular classification, particularly in cancer prognosis. Similarly, Wang et al.^8^ highlighted its role in decoding genetic signatures associated with drug response, using multi-omics profiles such as genomic mutations, copy number alterations, and gene expression to enhance drug repurposing models. Furthermore, Gong et al.^6^ underscored the need for advanced deep learning models to integrate multi-omics data effectively, as traditional methods often overlook correlations between patients and different omics layers.

**Fig. 3:**
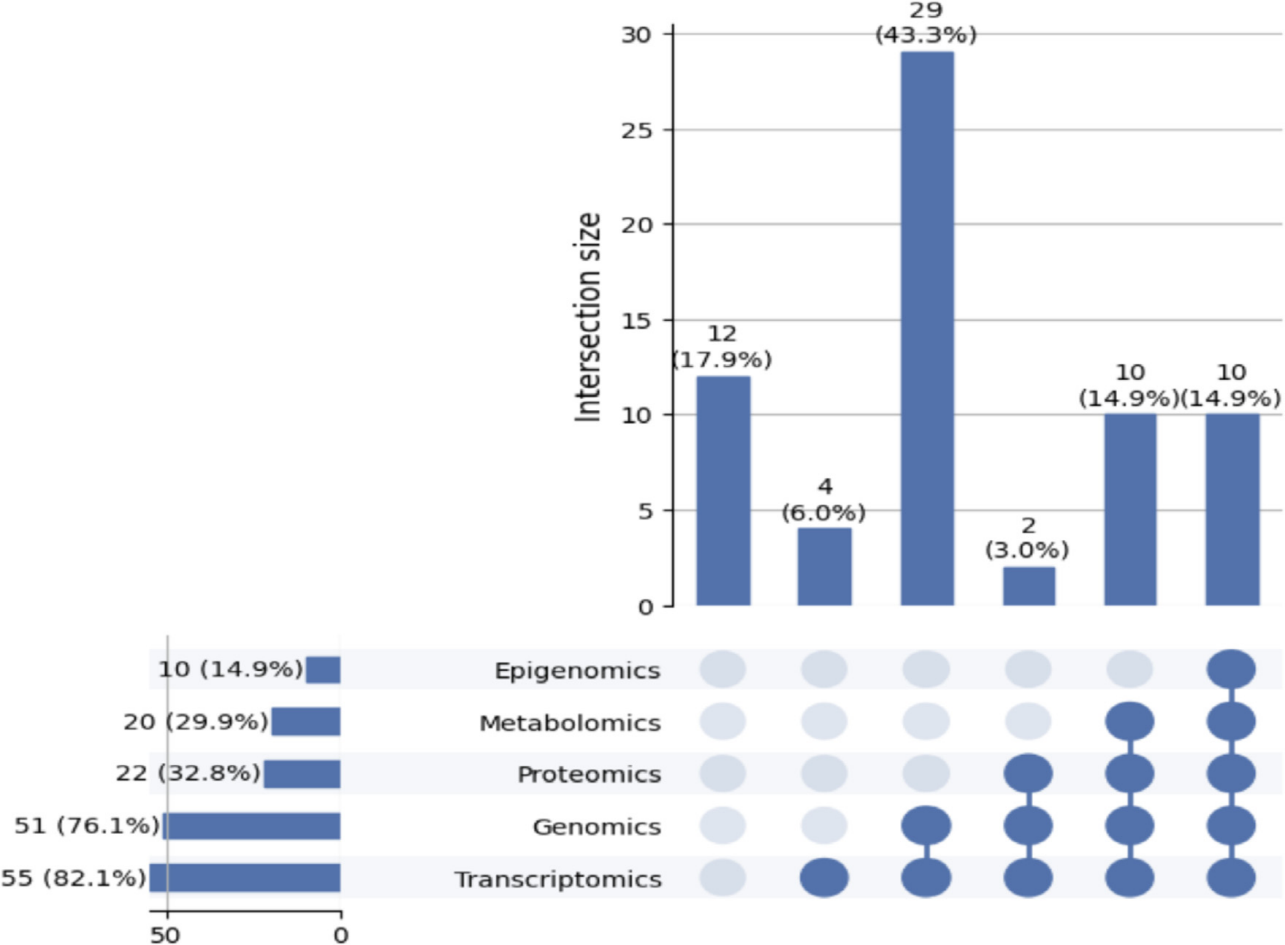
Number of publications using omics data type. Percentages reflect the distribution of omics data types within the 67 studies included in this review and may not necessarily represent broader publication trends across the omics research field.

According to the reviewed studies, Fig. 3 illustrates the frequency and cooccurrence of omics data types used in multi-omics analyses. Approximately 82.1% of the studies involved transcriptomics, and around 76.1% included genomics, underscoring their dominant role in integrative research. Proteomics appeared in about 32.8% of the studies, whereas metabolomics and epigenomics were included in approximately 29.9% and 14.9% of cases, respectively, reflecting a comparatively lower emphasis on these layers. Furthermore, the most frequent combination was the integration of transcriptomics and genomics, occurring in roughly 43.3% of the studies. This was followed by transcriptomics, proteomics, and metabolomics, with transcriptomics accounting for around 17.9% transcriptomics and metabolomics about 14.9%. These percentages indicate the distribution within the analyzed publications and may not accurately reflect the overall trends in the broader omics research field.

### Genomics

Genomics investigates the structure, function, and variation of the genome. It focuses on genetic alterations such as single-nucleotide polymorphisms (SNPs), insertions–deletions, structural variants, and copy number changes.^9^ High-throughput sequencing methods, such as whole-genome sequencing (WGS), whole-exome sequencing (WES), and genome resequencing, enable the identification of these variants.^10^ Analytical approaches like genome-wide association studies (GWAS) and expression quantitative trait loci (eQTL) mapping further elucidate the relationship between genetic variation, gene expression, and phenotypic traits.

Moreover, advancements in next-generation sequencing (NGS) have significantly accelerated genomic analysis, enhancing our understanding of disease mechanisms and supporting the development of targeted therapies.^11^ Third-generation sequencing technologies, including nanopore and single-molecule real-time methods, offer long-read capabilities, albeit with higher error rates.^9^

### Epigenomics

Epigenomics investigates reversible regulatory mechanisms that influence gene expression without altering the DNA sequence. These include DNA methylation, histone modifications, and chromatin architecture.^12^ Together, these layers of regulation contribute to chromatin accessibility, DNA modifications, and nucleic acid interactions, offering critical insights into enhancer function and the dynamic control of gene activity.^13^

To investigate these processes at higher resolution, technologies such as single-cell Assay for Transposase-Accessible Chromatin using Sequencing have emerged. This approach enables profiling of chromatin accessibility at the single-cell level, facilitating the characterization of cell-type-specific regulatory landscapes.^14^ Therefore, it supports a deeper understanding of gene regulation in heterogeneous tissues and advances non-invasive diagnostics by linking epigenetic states to environmental and genetic factors.

### Transcriptomics

Transcriptomics quantifies RNA expression across different conditions, providing essential data for computational and deep learning analyses.^10^ In particular, RNA sequencing (RNA-seq) is widely adopted due to its sensitivity and accuracy, whereas single-cell RNA-seq (scRNA-seq) enables cell-level resolution of expression patterns.^15^^,^^16^ Given the complexity of scRNA-seq data, analytical steps such as normalization, dimensionality reduction, and trajectory inference are required to extract biologically meaningful signals.^17^^,^^18^

Furthermore, spatial transcriptomics further extends this analysis by retaining spatial context within tissues. Through methods like in-situ sequencing and spatial barcoding, it supports the investigation of regional gene expression and cell–cell interactions in developmental and pathological contexts.^19^^,^^20^

### Proteomics

Proteomics focuses on the large-scale characterization of proteins, including their expression levels, localization, post-translational modifications, and interactions.^21^ Techniques such as mass spectrometry, often coupled with interaction assays like phage display or yeast two-hybrid, are commonly used to identify protein–protein interactions and regulatory modifications.^22^ Moreover, the dynamic nature of the proteome, which is shaped by factors such as cell type, functional state, and external stimuli, adds complexity to the analysis. However, this complexity also offers valuable insights for biomarker discovery and disease mechanisms.^21^^,^^23^

### Metabolomics

Metabolomics involves the large-scale profiling of small molecule metabolites, providing insight into metabolic phenotypes and disease-associated biochemical alterations.^9^^,^^24^ Furthermore, recent advances in single-cell metabolomics (SCM), particularly mass spectrometry-based techniques, are enhancing resolution at the cellular level. SCM enables the study of cellular heterogeneity and, when integrated with other omics layers, contributes to understanding differentiation, disease mechanisms, and therapeutic targets.^25^

**Fig. 4:**
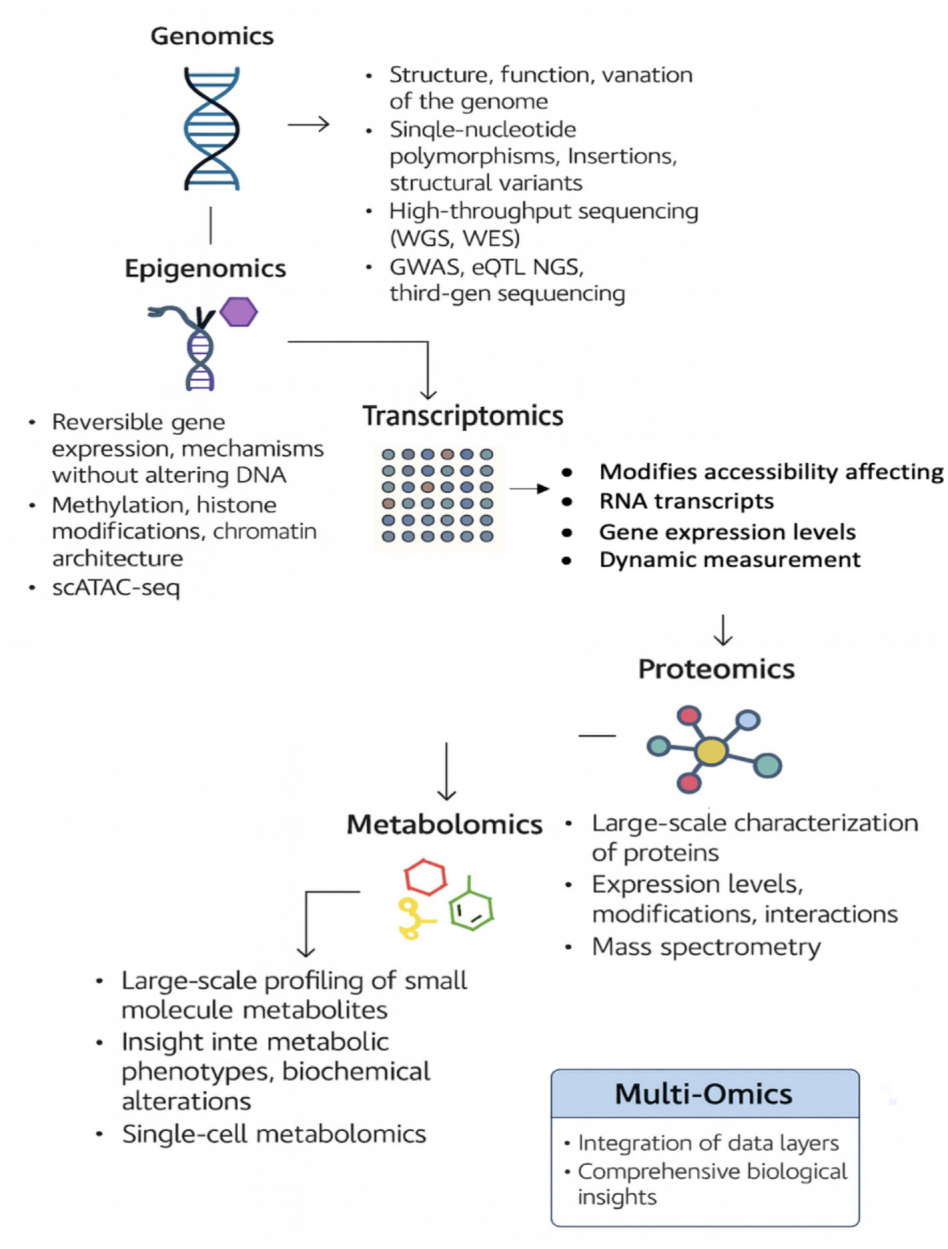
Overview of omics data.

Overall, Fig. 4 depicts that omics data types deliver a comprehensive understanding of biological systems by analyzing different molecular layers. Genomics explores DNA sequences, genes, and regulatory regions that influence cellular functions, whereas epigenomics studies chemical modifications like DNA methylation that regulate gene expression and impact development and diseases. Transcriptomics examines RNA transcripts to assess dynamic gene expression, proteomics investigates protein abundance and interactions, and metabolomics focuses on small-molecule metabolites, all of which interconnect as multi-omics layers to reveal complex biological mechanisms.

### Multi-omics data preprocessing and challenges

Multi-omics data integration requires omics-specific preprocessing strategies to ensure compatibility and minimize technical noise before applying deep learning. Table 1 summarizes multi-omics data preprocessing and the associated deep learning challenges. In genomics, variant calling and copy number profiles are often normalized and transformed into spatial formats for CNN input. Similarly, RNA-seq data undergoes filtering, normalization, and batch effect correction, utilizing tools like CIBERSORT for immune cell deconvolution. Furthermore, epigenomic preprocessing involves filtering low-quality or cross-reactive CpG probes and applying normalization to DNA methylation data to correct for technical variability. In addition, proteomics data are normalized and imputed before integration. In particular, metabolomics workflows employ log-normalization and feature harmonization before merging with other omics data.

**Table 1:** Multi-omics data preprocessing and deep learning challenges.

Omics	Raw data	Preprocessing steps	Feature types	DL challenges	Study
Epigenomics	Beta values from methylation arrays, beta values from sequencing	Filtering unreliable probes, integration into image space, signal smoothing	Histone marks, chromatin accessibility, DNA methylation	Data sparsity, varying feature sets across datasets	26, 27, 28, 29, 30
Genomics	Copy number alteration profiles, copy number profiles from segmented data	Normalization, conversion to image color channels	Copy number variation profiles, mutation data	Data heterogeneity, missing modalities, integration complexity	26, 27, 28
Metabolomics	Metabolite concentration matrices, processed metabolite profiles	Log normalization, standardization, integration with microbiome features	Metabolite concentrations, normalized intensity values	High missingness, chemical diversity, batch effects, inter-individual variability	^28^^,^^31^^,^^32^
Proteomics	Mass spectrometry-based peptide intensity matrices, RPPA data	Peptide identification, quantification, normalization, imputation	Protein abundance levels, protein expression levels	High missingness, dynamic range variation, low reproducibility	^27^^,^^28^^,^^33^
Transcriptomics	Gene expression matrices, RNA-seq count matrices	Adapter trimming, alignment, expression quantification, batch correction	Gene expression levels, differential expression	Batch effects, platform variability, temporal inconsistency	^26^^,^^28^^,^^33^

However, despite these preprocessing efforts, challenges such as high dimensionality and data sparsity remain. Therefore, there is a pressing need for robust methods for feature selection, dimensionality reduction, and integration to effectively manage the diversity of omics data and address missing data patterns.

### Image-based transformation methods for multi-omics data

Converting non-image omics data into image formats empowers the application of CNNs for classification and feature finding. In Table 2, methods such as DeepInsight, SurvCNN, Fotomics, and OmicsMapNet applied techniques like t-SNE, FFT, and treemaps to restructure data spatially, achieving accuracies ranging from 75% to 99%. These results highlight the potential of image-based transformations to improve predictive modeling in omics analysis.

**Table 2:** Studies apply techniques to transforming omics data into images.

Study	Purpose	Techniques apply	Model accuracy (%)
^5^	Utilization of CNNs for non-image data. Improved feature analysis. Enhanced classification accuracy.	t-SNE, kernel PCA to project features onto a 2D Cartesian plane. The 2D feature coordinates are then converted into pixels on a grid using the convex hull algorithm.	Classification accuracies across various datasets for RNA-seq data: 99%
^34^	Convert numerical omics data into structured images for CNN-based survival prediction.	t-SNE, UMAP-based spatial structuring	SurvCNN: 0.84–0.85%
^14^	To convert single-cell omics data into images for cell-type classification.	The fast Fourier transform (FFT) helps to identify patterns and periodicities within the data. The mapped features are framed using a convex hull algorithm	Average classification accuracy 90%
^35^	Transforms high-dimensional omics data into 2D treemap images, enabling DCNNs to classify tumor grades and discover phenotype-specific biomarkers.	A treemap, which is a space-filling visualization technique, using KEGG BRITE functional hierarchy.	Classification accuracy of 75.09% for tumor grading.

### Comparative overview of key image transformation methods

Different methods structure omics data into image formats in various ways. OmicsMapNet^35^ utilizes KEGG-based treemaps to group genes by their functions, supporting pathway-level interpretability but limiting flexibility. DeepInsight^5^ employs dimensionality reduction techniques such as t-SNE and kernel PCA to spatially arrange features based on similarity. This approach enables CNN-based classification with minimal preprocessing. SurvCNN^34^ adopts a similar method, using spatial layouts for survival analysis. Fotomics^14^ converts expression vectors into frequency-domain signals using FFT, which is advantageous for analyzing time-series and single-cell omics data, although it is less interpretable from a biological perspective.

Comparative studies^5^^,^^14^^,^^34^, ^35^, ^36^ indicate that DeepInsight and Fotomics often provide strong classification performance, whereas OmicsMapNet excels in interpretability. Each method is suited to different scenarios based on dataset type, noise level, and analytical objectives.

Techniques used in Table 2 are fundamental computational tools that facilitate the visualization, structural interpretation, and pattern recognition of complex, high-dimensional datasets across various analytical domains. The following are concise details of transformation techniques:

• *t-SNE.* t-distributed stochastic neighbor embedding (t-SNE) is a non-linear dimensionality reduction technique used for the visualization of high-dimensional biological data, including transcriptomic profiles derived from RNA-seq experiments. t-SNE models pairwise similarities between data points through probabilistic distributions. It minimizes the Kullback–Leibler divergence between these similarities in the original high-dimensional space and those in a corresponding low-dimensional embedding. The method employs a heavy-tailed Student's *t*-distribution in the low-dimensional space to preserve both local and global data structures, allowing for the identification of biologically relevant clusters. Due to the non-convex nature of its cost function and its sensitivity to hyperparameters such as perplexity, learning rate, and iteration count, careful parameter tuning and multiple evaluations are required to ensure reliable and interpretable results.^37^
• *Kernel PCA.* Kernel PCA (KPCA) is an advanced dimensionality reduction technique suitable for uncovering non-linear structures in high-dimensional datasets, such as RNA-seq data. It extends traditional PCA by projecting original input data into a high-dimensional feature space using kernel functions, commonly Gaussian kernels, to capture non-linear relationships implicitly.^38^ The technique has demonstrated strong performance on both artificial data distributions and practical datasets, such as handwritten digit recognition and breast cancer cytology classification tasks.^39^ Moreover, KPCA involves projecting original data onto a high-dimensional reproducing kernel Hilbert space through non-linear mapping and applying linear PCA within this transformed space.^40^ A significant advantage of this method is that it effectively captures non-linear relationships without explicitly computing coordinates in the high-dimensional space, relying instead on kernel methods and linear algebra to solve the eigenvector problem. Therefore, non-linearities inherent in real-world processes, KPCA has become a valuable tool for feature extraction, dimensionality reduction, and fault detection, particularly in industrial applications involving complex, non-linear systems.
• *UMAP.* Uniform manifold approximation and projection (UMAP) is a dimensionality reduction technique established on manifold learning, Riemannian geometry, and algebraic topology. It delivers a flexible and scalable approach for embedding high-dimensional data into lower-dimensional spaces, effectively preserving both local and global data structures. Its adaptability to various embedding dimensions makes it a versatile tool for general-purpose dimensionality reduction in machine learning.^41^ Furthermore, there are several variants of UMAP that cater to specific needs. For instance, DensMAP is designed to preserve data density, whereas parametric UMAP focuses on generating embeddings based on deep learning techniques.^42^ Another notable variant is progressive UMAP, which excels at handling streaming data and out-of-sample scenarios.^43^ This variant supports incremental projections, allowing for rapid approximations and facilitating interactive data analysis. In the context of RNA-seq, especially single-cell RNA-seq, to reduce high-dimensional gene expression data into low-dimensional space for visualization and analysis. It models local relationships among cells in high-dimensional space and preserves these in a lower dimension, enabling the identification of cell types, states, and trajectories. UMAP effectively captures complex biological structures and is integrated into standard scRNA-seq pipelines such as Seurat and Scanpy.^41^^,^^44^
• *FFT.* The fast Fourier transform (FFT) is an efficient algorithm for computing the discrete Fourier transform (DFT), enabling the conversion of data from the spatial domain to the frequency domain.^45^ However, it is used as a signal processing technique involving transforming raw data, for example, time-series signals and gene-expression vectors, from the time or spatial domain into the frequency domain to uncover hidden patterns such as periodicities or structural similarities. It has been examined in a study^14^ that the Fotomics framework, FFT is applied to RNA-seq data by treating each gene expression vector from scRNA-seq as a signal. This method induces complex numbers comprising real and imaginary parts, which are then mapped onto a two-dimensional (2D) Cartesian plane. Each gene is assigned coordinates based on its transformed values, allowing spatial representation of expression relationships across features. These coordinates are used to generate 2D images, encoding both gene expression intensities and inter-feature associations for downstream analysis using CNNs.
• *Treemap.* Treemaps are a hierarchical visualization method that uses space-filling rectangles to represent nested structures. Each rectangle's size and position reflect quantitative data and hierarchical relationships.^46^ Originally designed for efficient visual representation of tree-like data, treemaps have been adapted for bioinformatics to interpret complex omics datasets.^47^ Additionally, treemaps have practical applications in analyzing RNA-seq data within clinical contexts, such as identifying activated pathways in pediatric cancer and interpreting individual patient profiles.^48^ By mapping gene expression or pathway activation data into hierarchical rectangular structures, treemaps facilitate intuitive exploration of functional enrichment and molecular patterns within RNA-seq datasets. In OmicsMapNet,^35^ RNA-seq data are transformed into treemap images by arranging genes according to the KEGG BRITE functional hierarchies, with each gene's expression represented by color. This process allows CNNs to learn phenotype-related patterns from both gene expression and functional context.

Overall, the discussed transformation techniques, t-SNE, Kernel PCA, UMAP, FFT, and treemap, effectively reduce and restructure high-dimensional RNA-seq data for analysis and visualization. Individually, the method employs unique mathematical principles, such as probabilistic modeling and signal processing, to reveal biologically meaningful patterns.

## Deep learning models for image-based disease classification

Transforming omics data into images enables CNNs to capture spatial patterns that are difficult to model with raw tabular inputs. Methods like DeepInsight,^5^ SurvCNN,^34^ and Fotomics^14^ restructure omics features into 2D layouts using t-SNE, UMAP, or FFT, achieving improved classification and survival prediction performance often with 4–7% gains in AUC or accuracy compared to MLP-based and other traditional classifiers. These approaches also support transfer learning and visual interpretability.

Whereas graph-based models like MOFNet^63^ and MOGONET^49^ can perform comparably without image transformation, they rely on predefined inter-omics relationships. In contrast, image-based methods generalize well across omics types and data sizes. Thus, despite the added preprocessing, image conversion is often beneficial for high-dimensional, sparse, or small-sample omics datasets, where spatial structure can enhance learning.

This section explains the top five techniques from Table 3. Image analysis has been transformed by CNNs, AEs, and SVMs, which enable efficient feature prediction, reconstruction, and classification. GCNs and GNNs further enhance analysis by modeling complex spatial and relational structures, making them especially powerful for advanced imaging tasks.

• *CNN.* Neural networks were identified as the leading model for image analysis due to their direct learning capabilities of ConvNets and classification efficacy.^78^ CNNs are a class of deep learning architectures that demonstrate exceptional performance in image segmentation and classification. These architectures excel in hierarchical feature extraction via multiple backpropagation layers, allowing for local pattern recognition and computational efficiency achieved through weight sharing and pooling mechanisms. Furthermore, CNNs have proven particularly effective in transforming non-image data into visual representations for analysis.^52^ This capability extends to multi-omics data integration, significantly benefiting cancer research and biomarker discovery.^28^^,^^32^ CNNs excel in multi-omics analysis by facilitating the conversion of complex biological data into image representations, which, in turn, enables superior classification accuracy and robust performance in cancer subtype prediction and survival estimation.^26^^,^^34^ In the end, advancements in CNNs are crucial for improving the understanding and classification of complex biological data.
• *AE.* Autoencoders (AEs) are neural networks used for unsupervised tasks such as dimensionality reduction and anomaly detection. They are essential in multi-omics research for learning compact, noise-resistant representations from diverse, high-dimensional datasets. For example, Chaudhary et al.^53^ demonstrated that AE-based models can stratify hepatocellular carcinoma patients into subgroups with significantly different survival profiles, validated across five independent cohorts. In addition, Lv et al.^56^ used an AE framework for survival prediction in colon adenocarcinoma and showed it outperformed PCA, NMF, and t-SNE in clustering robustness and clinical relevance. The DeepProg framework highlights the effectiveness of AE-based ensemble models in pan-cancer prognosis, utilizing RNA, miRNA, and methylation data across^32^ cancer types.^58^ Advanced architectures like denoising AEs accurately estimate gene expression from methylation and CNV data.^13^ The MOCAT model, which combines auxiliary classifiers with attention-enhanced AEs, shows improved disease classification across various cancer types.^54^ Additionally, AEs aid in integrating histopathology images and genomic features, enhancing biomarker discovery in cancer imaging and omics data.^27^ ConcatAE and CrossAE are deep learning AEs for integrating multi-omics data in survival analysis. ConcatAE processes each omic modality separately, concatenating its hidden features to retain unique insights. Whereas CrossAE reconstructs one modality's features from another to achieve a consensus, creating a modality-invariant representation.^70^ Finally, AEs reveal complex patterns in multi-omics and image-rich biomedical data, serving as a powerful tool for predictive modeling in precision oncology.
• *SVM.* The support vector machine (SVM) is a supervised machine learning algorithm known for its effectiveness in classifying linearly separable data, and it has arisen as an effective tool in multi-omics data integration. For instance, an SVM trained on LASSO-selected genes in cutaneous melanoma demonstrated remarkable efficacy by distinguishing risk subtypes with over 95% accuracy, utilizing multi-omics features extracted through deep learning AEs.^31^ Furthermore, the DeepProg framework showcased the strengths of SVMs within an ensemble model that synergistically integrated deep learning and machine learning techniques, achieving significant advancements in prognosis prediction across^32^ cancer types and outperforming other integration methods.^58^ In the context of liver cancer, SVMs have proven instrumental in robust risk classification and survival prediction when combined with multi-modal omics inputs such as RNA-seq, miRNA, and methylation data.^53^ Similarly, the DeepKEGG model employed interpretable SVM-based architectures, enhancing predictions of cancer recurrence and facilitating biomarker discovery.^32^ Research has shown that SVM is effective in capturing phenotype variations and achieving high AUC values for disease classification, particularly in liver disease diagnosis and COVID-19 prognosis.^59^, ^60^, ^61^ Comprehensive cancer studies highlight SVMs' ability to uncover subtype-specific molecular patterns, especially when combined with early or late data fusion techniques.^9^ These findings emphasize the adaptability, precision, and interpretability of SVM in multi-omics pipelines, particularly when integrated with image-based features and deep learning.
• *GCN.* Graph convolutional networks (GCNs) have become a major deep learning architecture for analyzing multi-omics data, particularly where image features or graph-structured biological data are involved. Traditional CNNs are designed for grid-like data, such as images, whereas GCNs are specifically crafted to process non-Euclidean data through graph structures. For example, GCN-based frameworks like MOGONET and MORONET have demonstrated superior performance in combining mRNA, DNA methylation, and miRNA data for cancer subtype classification while also identifying key biomarkers.^49^^,^^64^ Furthermore, MOFNet leverages GCNs to model inter-omics label correlations for enhanced disease classification.^63^ A GCN model integrating multiple omics modalities in non-small cell lung cancer achieved a macro F1-score of 93.7%.^66^ Collectively, these findings validate GCNs as a foundational approach for image-enhanced multi-omics analysis, enabling interpretable, high-resolution insights into disease mechanisms.
• *GNN.* Graph neural networks (GNNs) are a transformative class of deep learning models that are able to examine complex biological systems, including integrating images and multi-omics data. Recent studies have demonstrated the strength of GNNs across a variety of applications. For example, in stroke research, a knowledge graph-based GNN framework effectively integrated mRNA, miRNA, circRNA, and methylation data for accurate etiology classification and biomarker discovery.^69^ In the context of chronic obstructive pulmonary disease (COPD), convolutional GNNs (ConvGNNs) were shown to outperform conventional classifiers by combining transcriptomics and proteomics with protein–protein interaction networks, with additional interpretability achieved via SHAP analysis.^33^ Furthermore, the MPK-GNN model effectively incorporated multiple biological priors to enhance molecular subtype prediction in cancer, demonstrating improved performance over standard multi-view and deep learning methods.^68^ Other work, such as GCFANet, introduced a GNN-based cross-modal feature aggregation network that combined graph-based structure learning with transformer modules to improve drug response prediction from multi-omics data.^73^ In breast cancer survival analysis, integrating complementary omics features using deep learning and contrastive mechanisms significantly boosted predictive accuracy.^70^ Further supporting these advances, reviews and practical implementations highlight GNNs' critical role in integrative microbiome research, enabling data fusion across metagenomics, metabolomics, and transcriptomics to support personalized therapeutic strategies.^71^^,^^72^ These studies underscore GNNs as robust, scalable, and interpretable tools that are redefining the standards for image-enhanced multi-omics analysis in precision medicine.

**Table 3:** Top 10 techniques in prior studies.

Technique	Article count	References
Convolutional neural networks (CNN)	13	^5^^,^^9^^,^^10^^,^^12^^,^^14^^,^^26^, ^27^, ^28^^,^^32^^,^^34^^,^^50^, ^51^, ^52^
Autoencoder (AE)	13	^7^^,^^9^^,^^12^^,^^13^^,^^18^^,^^27^^,^^53^, ^54^, ^55^, ^56^, ^57^, ^58^
Support vector machine (SVM)	10	^9^^,^^31^^,^^32^^,^^53^^,^^58^, ^59^, ^60^, ^61^, ^62^
Graph convolutional networks (GCN)	8	^18^^,^^49^^,^^57^^,^^63^, ^64^, ^65^, ^66^, ^67^
Graph neural network (GNN)	7	^33^^,^^68^, ^69^, ^70^, ^71^, ^72^, ^73^
Random forest (RF)	6	^10^^,^^59^, ^60^, ^61^, ^62^^,^^74^
Deep neural network (DNN)	5	^10^^,^^59^^,^^61^^,^^62^^,^^74^
Multilayer perceptron (MLP)	5	^13^^,^^50^^,^^61^^,^^62^^,^^68^
Variational autoencoder (VAE)	5	^18^^,^^72^^,^^75^, ^76^, ^77^
Recurrent neural network (RNN)	3	^11^^,^^51^^,^^66^

Overall, the last decade has witnessed significant advancements in multi-omics integration driven by deep learning. From the corpus analyzed, it is evident that CNNs, AEs, GCNs, and deep neural networks (DNNs) are among the most frequently used techniques. These methods enable robust dimensionality reduction and feature extraction across high-dimensional omics data.

## Model evaluation techniques

**Fig. 5:**
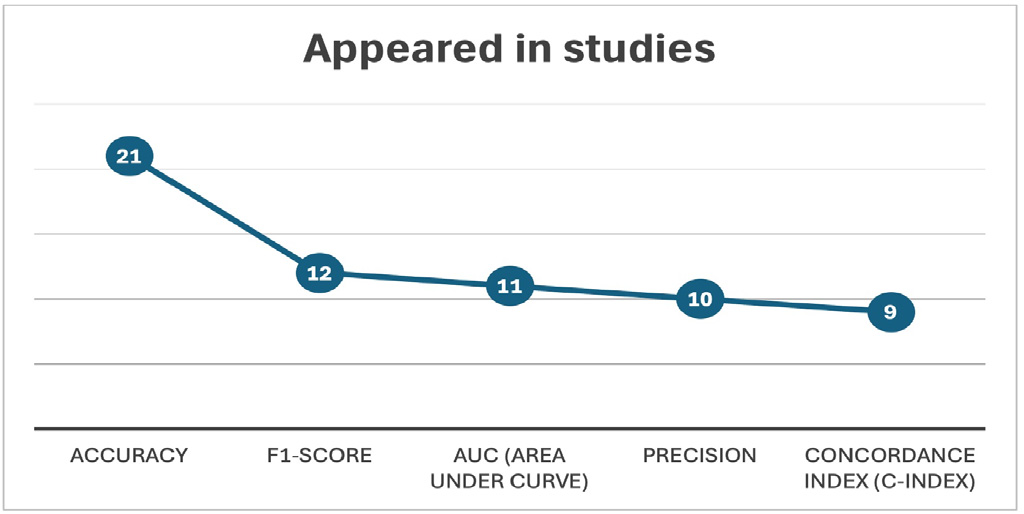
Common model evaluation metrics reported in the reviewed multi-omics deep learning studies (Table 3).

Evaluating deep learning models for multi-omics data presents distinct challenges, such as high dimensionality, small sample sizes, class imbalance, and the need for interpretability in clinical contexts. However, choosing appropriate evaluation strategies remains critical for robust and clinically meaningful modeling. Fig. 5 summarizes the most frequently reported evaluation metrics across the reviewed studies. Whereas these standard metrics remain useful, their application in omics contexts requires careful consideration.

### Accuracy

Accuracy measures the proportion of correctly predicted instances among all predictions:

(1) $$\text{Accuracy}=\frac{\mathrm{TP}+\mathrm{TN}}{\mathrm{TP}+\mathrm{TN}+\mathrm{FP}+\mathrm{FN}}$$

Although widely used, accuracy can be misleading in imbalanced datasets common in disease classification scenarios, where high accuracy may not reflect performance on minority classes.^27^^,^^32^

### F1-score

F1 score balances precision and recall using the harmonic mean, making it more suitable than accuracy for evaluating performance on imbalanced data:

(2) $$F1-\mathrm{score}=2\times \frac{\mathrm{Precision}\times \mathrm{Recall}}{\mathrm{Precision}+\mathrm{Recall}}$$

Frequently applied in multi-omics classification to ensure reliable detection of underrepresented disease subtypes.^30^^,^^54^

### AUC (area under the ROC curve)

AUC represents the probability that a randomly chosen positive instance ranks higher than a randomly chosen negative one. It is threshold-independent and particularly useful in binary classification:

(3) $$\mathrm{AUC}=\int_{0}^{1}\mathrm{TPR}\left(\mathrm{FPR}\right)\;\mathrm{dFPR}$$

However, in multi-class or highly imbalanced tasks, AUC must be interpreted cautiously.^60^

### Precision

Precision captures the proportion of correctly predicted positives among all positive predictions:

(4) $$\text{Precision}=\frac{\mathrm{TP}}{\mathrm{TP}+\mathrm{FP}}$$

This is particularly important in applications like biomarker discovery, where false positives can lead to costly downstream validation.^26^ Concordance index (C-index) evaluates the agreement between predicted risk scores and observed survival outcomes:

(5) $$C‐\mathrm{index}=\frac{\mathrm{Number of concordant pairs}}{\mathrm{Total comparable pairs}}$$

Extensively used in survival models for multi-omics integration, such as DeepProg, to assess time-to-event prediction accuracy.^56^^,^^58^

In future studies, incorporating complementary evaluation strategies such as calibration curves for reliability assessment, cross-validation schemes adapted to small sample sizes, and explainability tools such as SHAP or attention maps may further enhance model assessment in complex, multi-modal settings.

## Discussion

Multi-omics integration enhances disease understanding by combining complementary data across genomics, transcriptomics, proteomics, metabolomics, and epigenomics. As illustrated in Fig. 3, transcriptomics and genomics are the most frequently integrated, reflecting the maturity and accessibility of their platforms. In contrast, the lower use of proteomics, metabolomics, and epigenomics highlights ongoing challenges related to data quality, standardization, and computational integration. Each omics layer contributes unique biological insights, but their integration introduces considerable analytical complexity, necessitating meticulous preprocessing. Quality control is crucial for eliminating low-quality or noisy samples, followed by normalization to mitigate batch effects and ensure comparability across modalities. Feature selection methods such as PCA or mutual information filtering can be employed to reduce dimensionality while preserving essential biological variance. Imputation techniques address missing data, and alignment across datasets ensures that different omics layers are harmonized before subsequent transformation into image formats.

Transforming high-dimensional omics data into image-based representations enables the application of powerful deep learning architectures, particularly CNNs, which are specifically designed to exploit spatially structured inputs. These networks are specifically designed to effectively leverage spatially structured inputs. In addition, CNNs benefit from local spatial correlations within the data, thereby facilitating the hierarchical extraction of features that capture intricate interactions among genes, proteins, and metabolites.

Furthermore, given that raw omics data lack inherent spatial structure, various transformation methods are applied to project features into 2D formats. Additionally, in our recent work,^36^ we demonstrated how transforming multi-omics data into RGB image formats with each omics layer mapped to a separate color channel enhances the capacity of CNNs to learn from complementary data types. The RGB-layered representation captures inter-omic relationships more effectively and improves pattern recognition in classification tasks. In these formats, biologically or statistically related variables are spatially co-located, thereby effectively mimicking the spatial patterns typically observed in natural images.

As summarized in Table 2, transformation techniques such as t-SNE, Kernel PCA, UMAP, and FFT help preserve local or global feature relationships during this dimensional rearrangement. For example, t-SNE captures local structures and reveals meaningful clusters, whereas UMAP balances local and global topology. These methods help encode gene–gene correlations, pathway co-membership, or expression similarities into a format that can be efficiently processed by CNNs. Alternative techniques like treemaps use functional hierarchies such as KEGG pathways to create biologically interpretable image layouts, albeit at the risk of oversimplification.

After transformed, these image representations enable the use of image-based deep learning models for disease classification and subtype discovery, as detailed in Table 3. CNNs, in particular, excel at extracting discriminative patterns across multi-scale spatial regions, leading to high classification accuracy in various studies. AEs further support dimensionality reduction and feature learning, whereas GNNs provide a complementary approach that retains network-level topological structures. Together, these models demonstrate the practical value of image-based inputs: they not only make deep learning architectures applicable but also enhance pattern discovery, performance, and, in some cases, interpretability when compared to raw tabular input.

The evaluation of these models requires careful selection of performance metrics to ensure reliability and clinical relevance. As summarized in Fig. 5, accuracy remains the most commonly reported metric, yet its utility diminishes in the presence of class imbalance. The F1-score offers a more balanced measure by accounting for both precision and recall, making it especially suitable for imbalanced clinical datasets. The area under the receiver operating characteristic curve (AUC) provides a threshold-independent assessment of classification performance and is widely applicable across diverse classification tasks. Precision becomes particularly important in biomarker discovery applications where the cost of false positives is high. The C-index serves as a standard for evaluating survival models by measuring concordance between predicted and actual patient outcomes over time.

Despite the progress made, several critical challenges remain that must be addressed to fully harness the potential of image-based multi-omics integration. Data scarcity and heterogeneity continue to limit model robustness, especially when integrating underrepresented omics modalities or working with rare disease cohorts. High-dimensional data, combined with limited sample sizes, increase the risk of overfitting, emphasizing the importance of rigorous cross-validation, external cohort validation, and larger, harmonized datasets. Model interpretability remains a significant concern, particularly for complex deep learning architectures, such as GNNs and CNNs, which often function as opaque black boxes. Developing explainable AI techniques that reveal biologically meaningful features will be crucial for clinical acceptance. Furthermore, advanced graph-based models, while offering superior integration capabilities, present substantial computational demands that may hinder their broader adoption, particularly in resource-constrained research environments.

The studies summarized in Table 4 highlight the growing diversity of deep learning strategies for multi-omics integration. Graph-based models such as MOFNet, MOGONET, and MORONET have demonstrated high accuracy in disease classification and survival prediction by capturing complex inter-omic relationships. More recent architectures, including MPK-GNN and GCFANet, incorporate attention mechanisms and prior biological knowledge such as pathway topologies or protein–protein interaction networks to improve both predictive performance and interpretability. Simpler models, including SVM-based frameworks or ensemble approaches combining AEs with conventional classifiers, for instance, DeepProg, DeepKEGG, maintain robustness with lower computational overhead but may underperform when modeling non-linear cross-modal dependencies.

**Table 4:** Studies of deep learning models employ multi-omics data.

Study	Key contribution	Evaluation tools	Limitations	Performance
^70^	Focuses on deep learning-based multi-omics integration via concatenation autoencoder (ConcatAE) and cross-modality autoencoder (CrossAE)	Concordance index (C-index)	Limited sample size of TCGA-BRCA dataset, reliance on specific-omics modalities, lack of biological validation for biomarkers identified by models.	C-index of 0.641 ± 0.031.
^33^	Introduces a graph convolutional neural network (GCN)-based method (MoGCN) for classifying chronic obstructive pulmonary disease (COPD) using multi-omics data (transcriptomics, proteomics, metabolomics). The GNN leverages biological pathway information to define graph edges, improving biological interpretability.	Accuracy, AUROC (area under ROC curve), AUPRC (area under precision-recall curve)	Small sample size. Potential overfitting due to high dimensionality. Fixed graph structure may not generalize across datasets. Interpretability limited, despite the use of pathways	Accuracy: up to 80% AUROC: up to 0.88% AUPRC: up to 0.86%
^73^	A novel graph convolutional network (GCN)-based architecture that models both global omics features and cross-modal dependencies. It integrates multi-omics data by encoding features into a graph structure for better representation learning.	Accuracy, F1-score, AUROC	High complexity of modeling cross-modal dependencies. Computational cost. Generalizability across datasets not fully validated. Limited interpretability of GNN models	AUROC: up to 0.933% Accuracy: up to 0.884% F1-score: up to 0.891%
^68^	Proposes a novel GNN framework (MPK-GNN) that integrates multiple types of prior knowledge for improved multi-omics analysis. Combines different graph priors into a single model to enhance node embeddings and classification performance.	Accuracy, F1-score, AUROC	Combining multiple priors may introduce noise or redundancy. Complexity in interpreting contributions from different priors. Scalability and transferability to other diseases not verified	AUROC: 0.89% Accuracy: 0.85% F1-score: 0.84%
^69^	GNN-based method on knowledge graphs integrating.	Accuracy, F1-score, precision, recall	Limited sample size. Class imbalance across stroke etiologies. Lack of external validation. No experimental implementation of GNNs	Accuracy: 0.88% F1-score: 0.86% Precision: 0.89% Recall: 0.85%
^63^	Proposes MOFNet, integrating GCN-based module (SGO—Similarity Graph pOoling with structure learning) and VCDN for cross-omics label correlation modeling. GCN is used to extract omics-specific features via similarity-based graphs; it enhances interpretability and reduces feature redundancy.	Accuracy (ACC), F1-weighted, F1-macro	GCN may still retain irrelevant or redundant features if pooling is not optimal. Computationally more intensive due to multiple omics and tensor operations. Requires well-processed and aligned multi-omics data	ACC = 0.844%, F1-weighted = 0.852%, F1-macro = 0.832%
^67^	GCN is not used in this study. The article presents a framework combining Bayesian ridge regression (BRR) with iterative similarity bagging (ISB) to integrate multi-omics data for regression tasks such as clinical outcome prediction.	Concordance index (C-index), Pearson correlation	No exploration of graph-based methods like GCN. Model assumes linearity in high-dimensional interactions. Lack of interpretability in bagging procedure.	C-index around 0.68 for survival prediction. Pearson correlation.
^49^	Proposes MOGONET, a novel GCN-based method where separate GCNs are trained on individual omics (e.g., mRNA, DNA methylation, miRNA) to generate omics-specific embeddings. These are fused in the label space using view correlation discovery network (VCDN).	Accuracy, Macro F1-score, AUC	Omics-specific GCNs may not fully capture inter-omics dependencies. Performance sensitive to graph construction (e.g., k-nearest neighbor parameters). Limited external validation.	BRCA classification: Accuracy = 82.9%, Macro F1 = 0.774. GBMLGG: Accuracy = 95.9%. Demonstrated strong biomarker identification capability.
^64^	Introduces MORONET, an advanced framework that combines omics-specific GCNs with a view correlation discovery network (VCDN). Each omics data is modeled as a graph using patient similarity; GCNs extract high-level features, which are fused using VCDN for final classification.	Accuracy, Macro F1-score	Omics-specific GCNs do not model cross-omics interactions directly. High-computational complexity. Dependent on quality of similarity graph construction.	BRCA dataset: Accuracy = 83.6%, Macro F1 = 0.792. ROSMAP (AD vs CN): Accuracy = 83.7%, Macro F1 = 0.836.
^31^	The study uses SVM as one of the baseline models for performance comparison with their proposed deep learning method. SVM is applied after selecting top features from integrated multi-omics data (mRNA, miRNA, DNA methylation).	Concordance index (C-index), AUC, Kaplan–Meier survival curves	SVM shows relatively lower performance on complex, non-linear relationships. Sensitive to input feature scaling and dimensionality. Limited interpretability for biological insights compared to deep models.	SVM performance: Mean C-index = 0.656, Deep learning model outperformed SVM with mean C-index = 0.722.
^62^	SVM is employed as a baseline classifier for comparison with the proposed deep learning framework. It is applied separately to microbiome, metabolome, and combined data to evaluate classification performance.	Accuracy, precision, recall, F1-score	SVM performs sub-optimally on high-dimensional, non-linear multi-omics data. Limited adaptability to heterogeneous data types. Lower performance compared to integrated deep models.	SVM performance (combined data): F1-score: 0.712. Accuracy: 0.735. Deep learning model (MF-CNN): F1-score = 0.823, Accuracy = 0.843, outperforming SVM significantly.
^53^	SVM is used as a benchmark model for comparison. It is applied to each omics modality and to integrated data to evaluate survival prediction performance against deep learning methods.	Concordance index (C-index), log-rank test	SVM unable to model complex non-linear interactions in high-dimensional omics data. Lower predictive accuracy compared to deep integration approaches. Not well-suited for multi-modal data fusion.	SVM performance: Mean C-index = 0.627 on integrated data. DeepSurv (deep model) outperformed SVM with mean C-index = 0.719, showing clear benefit of deep integration for survival analysis.
^32^	SVM is employed as a baseline model to compare with the DeepKEGG deep learning framework. It is applied to evaluate classification performance using multi-omics data (gene expression, methylation, CNV).	Accuracy, AUC, F1-score	SVM struggles with modeling complex non-linear relationships across omics. Lower interpretability and performance for multi-modal integration. Performance sensitive to feature scaling and selection.	SVM performance: Accuracy = 0.743, AUC = 0.801. DeepKEGG significantly outperforms with Accuracy = 0.856, AUC = 0.902
^58^	SVM is used as a core component of the ensemble machine learning layer in DeepProg. After deep feature extraction via autoencoders, SVM (alongside logistic regression and random forest) is applied to build predictive models for patient survival.	Concordance index (C-index), log-rank *p*-value, Kaplan–Meier survival analysis	SVM performance depends on deep features and tuning. Less effective as a standalone model compared to ensemble. Interpretability of individual model components may be limited.	In ensemble setting (DeepProg): C-index = 0.77 (TCGA HCC), 0.73 (TCGA BRCA).
^59^	SVM used as part of a comparative machine learning suite to classify pathology phenotypes based on image and gene expression data. Found particularly effective in thyroid-Hashimoto's disease classification.	AUC (area under the curve), 5-fold cross-validation	Limited predictive accuracy for some pathologies; combining omics and image data did not always improve prediction; GTEx tissue images less detailed for some conditions	Best results: Thyroid-Hashimoto's (AUC = 0.96 with SVM on expression data), Liver congestion (AUC = 0.76 with SVM on expression data), other pathologies ranged from AUC = 0.57–0.84
^27^	Implements a multi-branch autoencoder (MBAE) architecture to fuse transcriptomics, epigenomics, and proteomics data for biomarker discovery and classification. AE branches separately encode each omics type and combine in a shared latent layer.	Accuracy, precision, recall, F1-score	Complex model structure increases training time. Sensitive to omics data preprocessing and balancing. Risk of overfitting due to many latent parameters	On TCGA datasets: Accuracy = 0.91, F1-score = 0.89.
^61^	SVM is reviewed as a widely used ML method in liver disease diagnosis, especially for binary classification tasks like fibrosis stage prediction and HCC detection. It is praised for its performance in high-dimensional but small datasets.	Accuracy, AUC, sensitivity, specificity (across cited studies)	Limited interpretability. Performance sensitive to kernel and parameter tuning. Often benchmarked in small-scale studies with limited generalizability.	Reported performances from reviewed studies: Fibrosis staging: AUC up to 0.92. HCC classification: Accuracy up to 94%, Specificity = 90%, Sensitivity = 93% (depending on dataset and features used).
^60^	SVM is highlighted as a key classification model used in various COVID-19 studies, particularly effective for distinguishing between infected and healthy individuals using transcriptomic and proteomic data.	Accuracy, AUC, precision, recall	SVM models require careful feature selection and kernel tuning. Potential overfitting with small COVID-omics datasets. Not inherently explainable; black-box issue for clinical adoption.	Across reviewed studies: Transcriptomics-based SVM models reported AUC up to 0.95, accuracy 90% in COVID-19.
^9^	SVM is reviewed as a classic ML model used for cancer subtype classification and biomarker discovery from multi-omics data. Cited as effective for small-sample, high-dimensional scenarios such as miRNA or mRNA expression classification.	Accuracy, AUC, precision, recall	Lacks scalability for complex, multi-layered omics integration. Susceptible to overfitting in high-dimensional data. Interpretability and feature relevance not inherent.	Reviewed studies report: SVM accuracy in cancer classification: 85–95% AUC values up to 0.96 in tasks like breast cancer subtype prediction.
^7^	The study utilizes a stacked autoencoder (SAE) to integrate multi-omics data (transcriptome, methylome, and proteome) and extract low-dimensional, informative features for survival analysis. AEs help denoise and compress high-dimensional data into a unified latent space.	Concordance index (C-index), Kaplan–Meier survival analysis, log-rank *p*-value	SAE performance depends heavily on architecture and training stability. Risk of overfitting on small datasets. Interpretability of latent features remains a challenge.	SAE-based model outperforms traditional methods: relapse prediction: C-index = 0.782
^55^	Utilized deep autoencoders to extract informative, low-dimensional features from multi-omics data (mutations, CNV, mRNA, methylation). These features were input into a deep neural network to predict IC50 drug response scores. AE facilitated effective dimensionality reduction and denoising.	Accuracy, AUC, FPR, precision, MSE, correlation coefficient	Limited to in vitro data from cell lines (not real clinical data). Generalizability may be reduced. Model interpretability not discussed. Dependent on omics data quality.	Outperformed SVM and Malik et al. model: Example—Docetaxel: Accuracy = 0.80, AUC = 0.82, Precision = 0.95. Best model MSE = 0.02, Correlation coefficient = 0.73.
^13^	Implements an autoencoder-inspired deep learning regression model to infer gene expression levels from methylation and CNV data. The encoder learns a compact representation of regulatory inputs that predict mRNA levels.	Pearson correlation coefficient, mean squared error (MSE)	Limited to modeling expression from only methylation and CNV. Not validated on external datasets. Model interpretability and biological insight are limited.	Best model (DLGR): Pearson correlation = 0.88, MSE = 0.016. Outperformed baseline linear and shallow models, showing the AE-based regression effectively captures regulatory patterns
^54^	Proposes MOCAT, an advanced AE framework that integrates multi-omics data (gene expression, miRNA, methylation) using an autoencoder enhanced with auxiliary classifiers. The auxiliary classifiers guide the latent space to be more discriminative for downstream classification.	Accuracy, F1-score, AUC	Model performance sensitive to classifier and encoder balance. High training complexity. Limited interpretability of deep features	Outperformed several baseline models: On BRCA dataset: Accuracy = 0.882, AUC = 0.921.
^56^	Developed a deep autoencoder model to integrate RNA-seq, CNV, and methylation data for survival prediction in colon adenocarcinoma. AE was used to compress high-dimensional data into a common latent representation for prognosis modeling.	Concordance index (C-index), Kaplan–Meier survival analysis	Small sample size (*n* = 151). Potential overfitting due to complex model. Interpretability of latent features not addressed.	C-index = 0.778 for AE-based model.
^51^	CNNs were used for radiomics analysis on imaging data (CT/MRI) to extract tumor phenotypes and spatial heterogeneity. These features were integrated with multi-omics data for outcome prediction in pancreatic cancer.	Accuracy, precision, recall, F1-score, AUC-ROC	Deep models (CNN/RNN) are black-boxes; interpretation is complex. CT imaging may miss full spectrum of tumor features. Dataset heterogeneity and size variation across cohorts.	TCGA dataset: Accuracy = 0.85, AUC = 0.88. ICGC: Accuracy = 0.82, AUC = 0.85. CPTAC: Accuracy = 0.78, AUC = 0.82.
^5^	Introduces DeepInsight, a method to convert non-image data into image format for effective CNN processing, leveraging CNN's spatial feature extraction capabilities.	t-SNE, PCA	Requires dimensionality reduction for very high-dimensional data. Performance dependent on image resolution. Conversion may lose detail due to overlapping features in small image sizes.	CNN via DeepInsight achieved: RNA-seq = 99%, Vowels = 97%, Text = 92%, Madelon = 88%, Ringnorm = 98%; all outperforming decision tree, AdaBoost, and random forest benchmarks
^52^	Enhances the original DeepInsight by using CNNs to process multiple image-like representations of tabular (non-image) data. This includes multi-view transformations (e.g., PCA, t-SNE) to generate spatial layouts for CNN input.	Accuracy, F1-score, precision, recall	Computationally expensive due to multiple image transformations. Performance may vary across datasets. Image encoding step can obscure interpretability.	Multi-representation DeepInsight with CNN achieved superior performance over baseline models on multiple UCI datasets, achieving >95% accuracy on most tested datasets
^14^	Proposed Fotomics, a FFT-based method to convert omics data into images for CNN classification, effectively mapping cell identities from single-cell omics.	CNN classification accuracy, F1-score; benchmarked against SVM, RF, KNN, DeepInsight	Limited to transcriptomic data; lacks exploration in unsupervised or trajectory-based analyses	Average accuracy across datasets: 90% (Raw), 80% (CPM normalized), 76% (MinMax normalized)
^26^	CNN was applied to RGB-colored 2D gene similarity network (GSN) maps derived from Isomap embeddings of gene expression, CNA, and DNA methylation data for breast cancer stage prediction.	Accuracy, precision, recall, F1-measure, AUC	Limited to integrating three omics due to RGB color encoding; potential generalizability issues to other omics types	Accuracy = 99.85%, Precision = 99.97%, Recall = 99.92%, F1 = 99.94%, AUC = 99.97%
^28^	CNNs were used to process circular images derived from multi-omics data to learn feature representations and identify cancer subtypes. The circular encoding enhances spatial relationships among omics features.	Accuracy, precision, recall, F1-score, AUC	Dependent on effective transformation of omics data to image space; potential information loss in encoding; requires high computation	Achieved accuracy = 0.963, Precision = 0.951, Recall = 0.948, F1-score = 0.949, AUC = 0.973
^34^	Developed SurvCNN, a CNN-based model using omics-to-image transformations to predict survival outcomes in discrete time intervals. Effectively integrates omics with image-based modeling for survival analysis.	C-index, Brier score, log-rank test, survival curves	Interpretation of learned filters is complex; omics-to-image transformation may introduce biases; sensitive to image resolution and encoding scheme	SurvCNN achieved superior performance: C-index = 0.83, Brier score = 0.12

Despite these advances, model interpretability, generalizability, and computational efficiency remain central challenges. Transformer-based architectures, largely underexplored in many reviewed studies, have recently gained traction for their ability to model long-range dependencies and perform cross-modal attention across diverse omics layers. Emerging transformer variants tailored to multi-omics tasks such as OmicsFormer, MOTR, and MultiViT have leveraged lightweight adapters and multiple instance learning frameworks to enable fine-tuning on limited datasets and accommodate weakly labeled clinical data. Furthermore, post-hoc attention visualization, feature attribution methods, and confidence calibration techniques are increasingly integrated into these models to enhance transparency and support clinical interpretation.

Future research should prioritize the development of interpretable and scalable transformer-based architectures capable of integrating high-dimensional, heterogeneous omics data. Embedding biological priors such as gene regulatory networks and curated pathway databases directly into model architectures may further improve biological plausibility. Additionally, techniques such as data augmentation, synthetic data generation via Generative Adversarial Networks (GANs), and transfer learning may help overcome sample size limitations, particularly in rare diseases. Hybrid modeling frameworks that combine deep learning with classical machine learning, like a random forests and SVMs, offer promising solutions for translational applications, where interpretability and deployment speed are critical. Ultimately, interdisciplinary collaboration will be key to translating these computational advances into clinically meaningful tools for precision medicine.

## Conclusion

This review underscores the emerging significance of transforming multi-omics data into image formats for disease classification. The fusion of computational image transformation techniques with deep learning architectures offers new avenues for deciphering complex molecular interactions underlying diseases. Whereas substantial progress has been achieved, key challenges remain, particularly regarding data heterogeneity, model generalizability, interpretability, and the need for larger, well-curated datasets. Future research should prioritize the development of more interpretable models and robust transformation pipelines that can accommodate diverse omics modalities while maintaining biological relevance. Furthermore, collaborative efforts integrating computational scientists, clinicians, and biologists are essential to ensure the translational impact of these methodologies. As these technologies continue to evolve, image-based multi-omics integration holds substantial potential to advance personalized medicine, improve diagnostic accuracy, and facilitate targeted therapeutic intervention.

## Declaration of competing interest

The authors declare that they have no known competing financial interests or personal relationships that could have appeared to influence the work reported in this article.

## Data Availability

The datasets generated and analyzed during the current study are available in the Figshare repository, accessible via the DOI: https://doi.org/10.6084/m9.figshare.29364566.v1.
